# The effect of early administration of rectal progesterone in IVF/ICSI twin pregnancies on the preterm birth rate: a randomized trial

**DOI:** 10.1186/s12884-020-03033-4

**Published:** 2020-06-09

**Authors:** Mona Mohamed Aboulghar, Yahia El-Faissal, Ahmed Kamel, Ragaa Mansour, Gamal Serour, Mohamed Aboulghar, Yomna Islam

**Affiliations:** 1 The Egyptian IVF center Maadi, 3, St. No. 161-Hadayek El-Maadi, 11431, Cairo, Egypt; 2 Department of obstetrics and Gynecology, Cairo University, Cairo, Egypt; 3 Cairo Fetal Medicine Unit, Cairo University, Cairo, Egypt; 4 Department of Obstetrics and Gynecology, Al Azhar University, Cairo, Egypt

**Keywords:** IVF/ICSI, Twins, Preterm birth, Rectal progesterone, First trimester scan, And randomized placebo controlled double blind study

## Abstract

**Background:**

The rate of multiple pregnancies in IVF/ICSI ranges from 20 to 30%. The incidence of preterm birth in multiple pregnancies is as high as 60% and is even higher in pregnancies conceived after IVF & ICSI. The effect of progesterone on prevention of preterm birth in twins is controversial. Our group has proven a positive effect in reduction of preterm birth, by starting progesterone from the mid-trimester, in exclusively IVF/ICSI singleton pregnancies but not twins. The purpose of our current study was to explore the effect of earlier administration of natural progesterone, in IVF/ICSI twin pregnancies starting at 11–14 weeks for prevention of preterm birth.

**Methods:**

This is a double-blind, placebo controlled, single center, randomized clinical trial. Women with dichorionic twin gestations, having an IVF/ICSI trial were randomized to receive natural rectal progesterone (800 mg daily) vs placebo, starting early from 11 to 14 weeks. They also received oral and vaginal antimicrobial agents as part of our routine treatment for vaginitis and urinary tract infection. They were randomized regardless of cervical length and had no previous history of preterm birth or known Mullerian anomalies. The primary outcome was spontaneous preterm birth rate before 37 weeks. The secondary outcome was; spontaneous preterm birth before 34, 32, 28 weeks and neonatal outcome.

**Results:**

A total of 203 women were randomized to both groups, final analysis included 199 women as 4 were lost to follow up. The base line characteristics as well as gestational age at delivery were not significantly different between the study and the placebo group (34.7 ± 3.6 vs 34.5 ± 4.5, *P* = 0.626). Progesterone administration was not associated with a significant decrease in the spontaneous preterm birth rates before 37 weeks (73.5% vs 68%, *P* = 0.551), before 34 (20.6% vs 21.6%, *P* = 0.649), before 32 (8.8% vs 12.4%, *P* = 0.46) & before 28 (4.9% vs 3.1%, *P* = 0.555) weeks.

**Conclusions:**

Rectal natural progesterone starting from the first trimester in IVF/ICSI twin pregnancies did not reduce spontaneous preterm birth.

**Trial registration:**

The trial was registered on 31 January 2014 at www.ISRCTN.com, number 69810120.

## Background

Multiple pregnancy remains one of the major complications of assisted reproductive techniques. A large percentage of ART-conceived babies are twin pregnancies, and multiple pregnancies are associated with a high rate of preterm birth, perinatal morbidity and mortality [1, 2] and hence it is considered to be the most serious complication of multiple pregnancy. Our published data showed a 64.8% preterm birth rate (before 37 weeks) in twin pregnancies [3].

The incidence of spontaneous preterm birth is higher for ART-conceived infants when compared to spontaneous pregnancies (31.2% vs 9.7%, before 37 weeks and in spontaneous very early preterm birth before 32 weeks; 5.2 vs 1.6, respectively) and in a more recent study 10.1% vs 5.5% before 37 weeks and 3.6% vs 2.2% before 34 weeks [4–6].

Prevention of spontaneous preterm birth in ART pregnancies is prioritized in an attempt to decrease prematurity complications. It was previously reported in several large randomized studies and meta-analysis that administration of progesterone resulted in a significant reduction in the rate of spontaneous preterm births in singleton pregnancies, when administered starting at mid trimester to all women, including those with a previous history of preterm birth as well as women with a short cervix [7–12]. Similarly, this positive effect of natural vaginal progesterone administration starting from mid-trimester was shown by our group in IVF singleton pregnancies regardless of their individual cervical length [3]. However, this same study did not reveal a significant reduction in preterm birth rate in IVF twins.

Controversy surrounds the use of progesterone in the prevention of preterm birth in twin gestation. Two large randomized controlled studies [13, 14] found no value in its use in twin pregnancy. A recent meta-analysis showed that in asymptomatic women with a mid-trimester sonographic short cervix, progesterone significantly reduced the risk of preterm birth before 33 weeks’ gestation by 31% [15].

Our present study aimed to explore the effect of earlier administration of natural rectal progesterone in IVF/ICSI twin pregnancies, starting at 11–14 weeks up to 37 weeks’ gestation, on spontaneous preterm birth rates.

## Methods

All pregnant women with dichorionic twins, who had not undergone cerclage, following an IVF/ICSI trial performed at the Egyptian IVF Center, Maadi, Cairo, Egypt, from January 2014 to July 2017 were counseled to participate in this study. They performed a first trimester scan and randomized women fulfilled the following Inclusion Criteria:

1) Non-smokers, 2) Normal fetal anatomy, 3) No uterine anomalies, 4) Dichorionic twins only, 5) No previous history of preterm birth, 6) Healthy pregnant women not suffering from medical disorders e.g. diabetes and hypertension, 7) No known allergy to progesterone.

Participants received 100 mg IM Progesterone injections (IBSA Egypt) daily for luteal phase support starting on the day of oocyte retrieval until the first viability ultrasound was performed at 7 weeks. Once fetal heart beats were confirmed they were advised to stop intake of progesterone. This was based on a previous study by our group which showed no significant difference in miscarriage rates in patients who stopped progesterone early as compared to those who continued to 10 weeks gestations [16].

To ensure eligibility of participants, all women enrolled in the study had a first trimester scan for risk assessment of Down syndrome in addition to an anomaly scan at 11–14 weeks of pregnancy. Dating was performed by calculation from the date of embryo transfer.

After the supervising obstetrician counseled the patient about the protocol and its aim and design, women who agreed to join the study signed a consent form. They were then randomized and began the suppositories immediately.

### Randomization

Randomization was done into either the Progesterone Group or the Placebo Group. This was performed by opening a numbered, sealed, opaque envelope, with an indication of either group (Progesterone and Placebo) at a ratio of 1: 1. The randomization was hand generated using the Microsoft Excel software which was executed by a third party (nurse) not involved in the trial. Progesterone and placebo suppositories were provided by the manufacturer (IBSA Egypt) in indistinguishable packaging, labeled with the patient’s code and the study name. The manufacturer was not involved in the writing of the protocol, study design or writing of the paper nor its submission. Suppositories were provided to the patient by the supervising nurse in sealed packages given once a month to cover the period before her next antenatal visit. On the next visit she returned the empty packages for confirmation of compliance.

### Intervention

The study group received natural progesterone suppositories of 400 mg, while the Placebo was composed of 126 mg hard fat, 36 mg Gelucire pellets, 0.2 mg Sorbic acid, 300 mg cocoa butter, and 195 mg purified water (Supplied by IBSA Egypt). They appeared identical. All suppositories were administered rectally twice daily starting from the time of randomization at 11–14 weeks until 37 weeks or delivery. Rectal administration was chosen as the social and cultural background of participating women showed that they have a great fear of repeated vaginal administration. In addition, we chose this route to minimize possible risks of vaginal infections from repeated prolonged periods of twice daily administration of vaginal suppositories.

Participants, supervising doctors, and nurses were unaware of the randomization and group allocation of the women. Only an independent secretary had the key code but had no access to the data nor to the participants. The blinding code was not broken until all data was collected from all participants, which took place a few weeks following delivery of the last patient. A long term follow up of the infants was not performed in this study.

Participants in both study and control groups received our routine antimicrobial agents given to twin pregnancy, for prophylactic and curative treatment of vaginitis and urinary tract infections. It is known that preterm premature rupture of membranes (PPROM) is the cause of approximately one third of preterm deliveries, and this rate is higher in our group of lower socioeconomic status patients [17].

All Patients received Clindamycin vaginal cream (2%) for 7 days each month as well as oral Amoxicillin as a single dose of 3 g once every month [18–20].

All women were followed up according to the routine antenatal care protocol of our institution. Every visit we confirmed adequate intake of the suppositories and took note of any reported side effects or adverse outcomes. Phone calls were also made to the patients to confirm compliance and monitor their condition. Collection of data was done following delivery by direct contact with the patient’s obstetrician and neonatologist. This included gestational age at delivery, neonatal condition, reporting of neonatal intensive care unit (NICU) admission, birth weight and congenital anomalies undetected during pregnancy.

### Study outcomes

Primary outcome measures: Spontaneous preterm birth before 37 weeks’ gestation.

Secondary outcome measures: Spontaneous preterm births before 34, 32 and 28 weeks.

Neonatal outcome; including: NICU admission, neonatal death.

### Relevant definitions

- Early fetal loss: fetal death between 11 and 22 weeks of gestational age [21]
- Late fetal loss: fetal death between 22 and 28 weeks of gestational age [21]
- Stillbirth: Fetal death after 28 weeks gestational age [21]
- NICU admission; neonatal admission to Intensive care for any reason other than neonatal jaundice
- Neonatal death; death up to 30 days after delivery

### Sample size estimation

Sample size calculation was based on a 20% reduction of the previously reported 65% preterm birth rate in twin pregnancies before 37 weeks of gestation [7].^.^ Calculation was done based on comparing 2 proportions from independent samples using Chi-square test wherein the α-error level was fixed at 0.05 and the power was set at 80%. Accordingly, the sample size was calculated to be 96 cases in each arm. Sample size calculation was done using PS Power and Sample Size Calculations software, version 3.0.11 for MS Windows (William D. Dupont and Walton D. Vanderbilt, USA).

### Ethics approval and consent to participate

All women read and signed an informed consent after full explanation of the study design and procedure. The study protocol was designed according to the CONSORT statement. Ethical approval was obtained on 15/11/2013 by our local Ethics Committee ‘Ethics and Research Committee of the Egyptian IVF Center’ headed by Professor Ibrahim Fahmy, (imfahmy@gmail.com) and received an IRB number of 3/2013. The trial was registered on 31 January 2014 at www.ISRCTN.com, number 69810120. There was no specific funding, the suppositories trial drug and placebo were provided by IBSA Egypt free of charge.

## Statistical methods

Data was statistically described in terms of mean ± standard deviation (± SD), or frequencies (number of cases) and percentages when appropriate. Comparison of numerical variables was done using Student *t* test for independent samples. The Chi-square test was used to compare categorical data between groups. A two-tailed *P* value < 0.05 was considered statistically significant. All statistical calculations were done using computer program SPSS (Statistical Package for the Social Science; SPSS Inc., Chicago, IL, USA) release 20 for Microsoft Windows (2006).

## Results

**Fig. 1:**
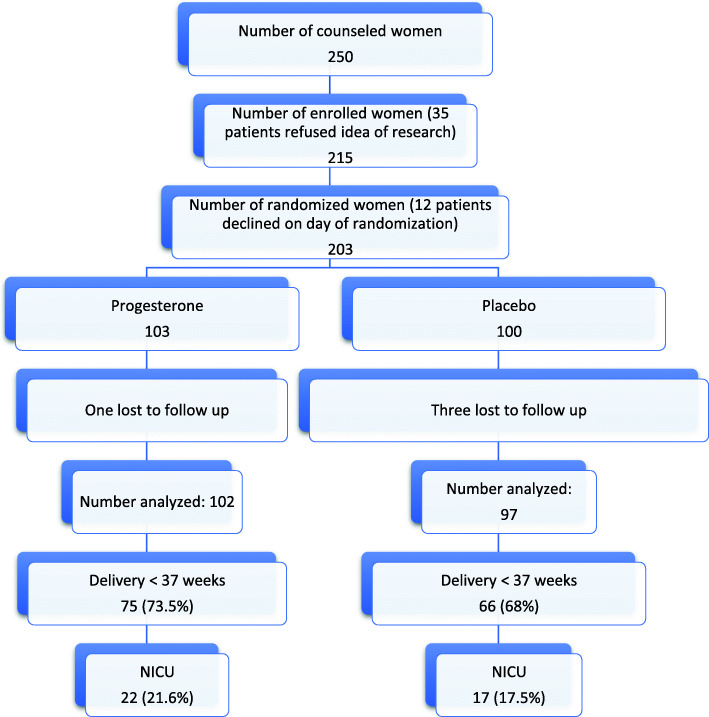
Flow Chart of Trial

We counseled 250 women to join the study: 215 were enrolled, and finally 203 signed the consent and were randomized (Fig. 1). The analysis was performed according to the intent-to-treat principle. Four women were lost to follow up, one in the study group and three in the placebo group. The study was completed, and analysis was done for 199 women. There was excellent compliance, all of the women adhering to twice daily suppository intake throughout the study period, except two women; one who stopped on her own at 24 weeks and a second who suffered bleeding esophageal varices and was for her safety recommended to stop any other medications. There were no reported side effects (such as thrombotic episodes) from the progesterone intake. All patients were followed up monthly by our obstetricians.

There was no significant difference between both groups with regards to age, BMI and gestational age at randomization, as shown in Table 1. All participating women were Egyptian. There was no significant difference in gestational age at delivery, term births, preterm births, or early fetal loss. Furthermore, there was no significant difference in preterm birth rates before 34, 32 and 28 weeks (Table 2).

**Table 1:** Baseline characteristics & gestational age at randomization

	Progesterone group	Control group	***P*** value
**(n)**	102	97	**–**
**Maternal age (years)**	29.5 ± 4.8	30 ± 4.6	0.498
**BMI (kg/m2)**	30.5 ± 5.8	30.1 ± 4.8	0.623
**Gestational age at randomization (weeks)**	12.7 ± 0.9	12.5 ± 1	0.341

Data is expressed either as a mean ± SD or n (%)

**Table 2:** Comparison of gestational age at delivery between both groups

		Progesterone group *N* = 102	Placebo group *N* = 97	
**Early fetal loss (n)**		2 (2%)	4 (4.1%)	0.372
**Gestational age at delivery (weeks)**		34.7 ± 3.6	34.5 ± 4.5	0.626
**Term births (n)**	**> 37 weeks**	25 (24.5%)	27 (27.8%)	0.594
**Preterm births (n)**	**< 37 weeks**	75 (73.5%)	66 (68%)	0.551
	**< 34 weeks**	21 (20.6%)	21 (21.6%)	0.649
	**< 32 weeks**	9 (8.8%)	12 (12.4%)	0.46
	**< 30 weeks**	5 (4.9%)	7 (7.2%)	0.516
	**< 28 weeks**	5 (4.9%)	3 (3.1%)	0.555

Data is expressed either as a mean ± SD or n (%)

Preterm births are all spontaneous births, except for two patients in the progesterone group who had iatrogenic termination < 37 weeks because of severe pre-eclampsia with no neonatal morbidity or mortality

Only two women were delivered electively preterm due to preeclampsia, the remaining women went into spontaneous preterm contractions or preterm rupture of membranes, otherwise were delivered electively by Caesarian section after 37 weeks.

Table 3 demonstrates the NICU admission, neonatal death and intrauterine fetal death, and neonatal survival rate in both groups. There was no significant difference between the study and control group in these parameters.

**Table 3:** Neonatal outcome in both groups

		Progesterone group *N* = 102	Placebo group *N* = 97	
**NICU admission (n)**	Single	9 (8.8%)	6 (6.2%)	0.726
	Both	13 (12.7%)	11 (11.3%)	
	Total	22 (21.6%)	17 (17.5%)	0.717
**Neonatal death (n)**	Single	7 (6.9%)	3 (3.1%)	0.404
	Both	6 (5.9%)	8 (8.2%)	
	Total	13 (12.7%)	11 (11.3%)	0.761
**Still birth (n)**	Single	2 (2%)	1 (1%)	0.513
	Both	–	1 (1%)	
**Neonatal survival rate (n)**	One	10 (9.8%)	4 (4.1%)	0.196
	Both	83 (81.4%)	80 (82.5%)	
	None	8 (7.8%)	13 (13.4%)	

Data is expressed either as a mean ± SD or n (%)

## Discussion

The wide use of assisted reproductive techniques such as IVF and ICSI as well as ovulation induction drugs for non-IVF cases has increased the rate of twin pregnancy worldwide [22, 23].

The overall reported incidence of twins following Assisted Reproductive Techniques (ART) in the latest ICMART (The International Committee Monitoring Assisted Reproductive Technologies) report was 20.9% [22]. The most recent data reported that approximately 33.9% of ART-conceived infants in the USA were twins, of which 62.4% were born preterm [4]. On the other hand, the preterm birth rate of twins following ART in Europe was measured at 48.8% [24].

The outcome of preterm neonates depends on the gestational age at birth and available resources of the Neonatology Unit caring for those infants [25].

It is of course noteworthy that care of premature neonates adds a huge financial burden on the health care provider and on the parents, especially when medical care is privately funded [26]. The objective of this study was to attempt a reduction in preterm birth rate in IVF/ICSI twin pregnancies. Specifically, we sought a significant reduction in twin preterm birth rates and the study was powered to detect a 20% reduction. While lower percentages of reduction were considered for a powered calculation, the 20% figure gave the most reasonable sample size and still maintained optimal significance.

This randomized placebo controlled double blind study did not show any benefit of giving natural rectal progesterone starting early on in pregnancy for prevention of preterm birth before 37 weeks in IVF/ ICSI twins. It is worth noting that the mean gestational age at delivery in both groups was +/− 34 weeks. This is earlier than that reported in most literature, which could be attributed to the different ethnicity of our studied group [27, 28]. In addition, pregnant women participating were all exclusively IVF pregnancies that are generally known to have a higher incidence of preterm birth [4]. Furthermore, a recent study by Saccone and colleagues [29] concluded that gestational age at delivery, for IVF-conceived twins, was earlier by about 1 week on average, compared with spontaneously-conceived twins, regardless of the cervical length measured in the second trimester.

The results of this study align with several previous publications [7, 13, 14]. However, it should be noted that all previous studies started progesterone administration at mid-trimester, while our study is unique in that it exclusively included IVF/ICSI twins starting from the first trimester.

Furthermore, this study used the rectal route instead of the vaginal route while all previously published studies used the vaginal route [7–10, 13, 14]. The rectal route approach has previously been studied and found to be as effective as the vaginal route [30–34], with reports of high serum progesterone levels detected at high doses [34]. We chose this route because our previous experience has shown more patient acceptability, comfort, and compliance with the rectal route [30]. It may also minimize any possibility of infection through the twice daily application of vaginal suppositories.

We have previously proven that progesterone is effective in reducing preterm birth [3] rates in singleton IVF/ICSI pregnancies starting from the mid-trimester, but did not find the same significance in twins, thus we postulated that starting earlier on in pregnancy might result in a significant reduction. We also chose the twins resulting from IVF/ICSI pregnancies because they are known to have a higher incidence of preterm birth [35] and are more closely monitored and followed up on. Moreover, we only included dichorionic twins as it is known that monochorionic twins are associated with higher miscarriage rates and other complications [36]. Other studies in the literature included both dichorionic and monochorionic twins [13].

Numerous trials have been published [7, 13, 14, 37–47] on the use of progesterone for prevention of preterm birth in twin pregnancies, but none of them were done exclusively on IVF/ICSI twins. Moreover, the results of these trials are controversial, as two big randomized placebo controlled trials found no significant reduction in preterm birth in twins before 34 weeks, after administration of progesterone regardless of cervical length [13, 14]. A more recent randomized controlled trial on twin pregnancies with short cervix (20–24 mm) showed that the administration of progesterone resulted in a significant reduction of preterm birth [40].

A recent meta-analysis showed that administration of vaginal progesterone to asymptomatic women with twin gestations and a sonographic short cervix in the mid trimester reduces the risk of preterm birth occurring before 30 to 35 weeks’ gestation [15]. Furthermore, the evidence in the above-mentioned study is of moderate quality with a moderate risk bias as a single study included in the meta-analysis produced most of the pooled effect [40].

In the latest report by the ICMART [22] including data from the years 2008–2010, single embryo transfer (SET) increased from 25.7 to 30% with a resulting drop in multiple twin births to 19.6% and triplets to 1.0%. Individual European countries with strict single embryo transfer policies such as Sweden have 76.9% of their transfers SET and a resulting 5.4% twin births [24]. Likewise, 84.9% of transfers in 2013 in Australia were SETs, resulting in a 5.5% twin delivery rate with persistently high 67.3% preterm birth rate [48]. This clearly shows that SET policy is the most successful preventive measure to reduce the problem of preterm birth in twins.

## Conclusions

Rectal progesterone starting from the first trimester is ineffective in reducing spontaneous preterm birth in IVF/ICSI twin pregnancies. As the risk of preterm birth is known to be high in twin pregnancies, the best approach in the management would be preventive. This can be achieved to a great extent by routine single embryo transfer after IVF/ICSI.

## Data Availability

The datasets used and/or analyzed during the current study are available from the corresponding author on request.

## Abbreviations

IM: Intramuscular
USA: United States of America
IVF/ICSI: In vitro fertilization/intracytoplasmic sperm injection
SET: Single embryo transfer
ART: Assisted Reproductive Techniques

## References

[1] Vogel JP, Torloni MR, Seuc A (2013). Maternal and perinatal outcomes of twin pregnancy in 23 low- and middle-income countries. PLoS One.

[2] Giuffre M, Piro E, Corsello G (2012). Prematurity and twinning. J Matern Fetal Neonatal Med.

[3] Aboulghar MM, Aboulghar MA, Amin YA, Al-Inany HG, Mansour RT, Serour GI (2012). The use of vaginal natural progesterone for prevention of preterm birth in IVF/ICSI pregnancies. Reprod BioMed Online.

[4] Sunderam S, Kissin DM, Crawford SB (2018). Assisted reproductive technology surveillance- United States, 2015. MMWR Surveill Summ.

[5] Cavoretto P, Candiani M, Giorgione V, Inversetti A, Abu-Saba MM, Tiberio F (2018). Risk of spontaneous preterm birth in singleton pregnancies conceived after IVF/ICSI treatment: meta-analysis of cohort studies. Ultrasound Obstet Gynecol.

[6] Rahu K, Allvee K, Karro H, Rahu M (2019). Singleton pregnancies after in vitro fertilization in Estonia: a register-based study of complications and adverse outcomes in relation to the maternal socio-demographic background. BMC Pregnancy Childbirth.

[7] Fonseca EB, Celik E, Parra M, Singh M, Nicolaides KH (2007). Fetal Medicine Foundation second trimester screening group. Progesterone and the risk of preterm birth among women with a short cervix. N Engl J Med.

[8] De Franco EA, O’Brien JM, Adair CD (2007). Vaginal natural progesterone is associated with a decrease in risk for early preterm birth and improved neonatal outcome in women with a short cervix: a secondary analysis from a randomized double-blind placebo-controlled trial. Ultrasound Obstet Gynecol.

[9] Hassan SS, Romero R, Vidyadhari D (2011). PREGNANT trial. Vaginal progesterone reduces the rate of preterm birth in women with a sonographic short cervix: a multicenter, randomized, double-blind, placebo-controlled trial. Ultrasound Obstet Gynecol.

[10] Romero R, Conde-Agudelo A, Da Fonseca E (2018). Vaginal progesterone for preventing preterm birth and adverse perinatal outcomes in singleton gestations with a short cervix: a meta-analysis of individual patient data. Am J Obstet Gynecol.

[11] Dodd JM, Jones L, Flenady V (2013). Prenatal administration of progesterone for preventing preterm birth in women considered to be at risk of preterm birth. Cochrane Database Syst Rev.

[12] Blackwell SC, Gyamfi-Bannerman C, Biggio JR (2018). PROLONG clinical study protocol: Hydroxyprogesterone Caproate to reduce recurrent preterm birth. Am J Perinatol.

[13] Rhode L, Klein K, Nikolaides KH (2011). Group. Prevention of preterm delivery in twin gestations (PREDICT):a multicenter, randomized, placebo-controlled trial on the effect of vaginal micronized progesterone. Ultrasound Obstet Gynecol.

[14] Norman JE, Marlow N, Messow CM (2016). Vaginal progesterone prophylaxis for preterm birth (the OPPTIMUM study) : a multicenter, randomized, double-blind trial. Lancet.

[15] Romero R, Conde-Agudelo A, El-Refaie W (2017). Vaginal progesterone decreases preterm birth and neonatal morbidity and mortality in women with a twin gestation and a short cervix: an updated meta-analysis of individual patient data. Ultrasound Obstet Gynecol.

[16] Aboulghar MM, Amin YM, Al-Inany HG, Aboulghar MA, GI MLMS, Mansour RT (2008). Prospective randomized study comparing luteal phase support for ICSI patients up to the first ultrasound compared with an additional three weeks. Hum Reprod.

[17] Medina TM, Hill DA (2006). Preterm premature rupture of membranes: diagnosis and management. Am Fam Physician.

[18] Lamont RF, Nhan-Chang CL, Sobel JD (2011). Treatment of abnormal vaginal flora in early pregnancy with clindamycin for the prevention of spontaneous prevention of spontaneous preterm birth: a systematic review and metaanalysis. Am J Obstet Gynecol.

[19] Smaill F, Vasquez JC (2007). Antibiotics for asymptomatic bacteriuria in pregnancy. Cochrane Database Syst Rev.

[20] Chu CM, Lowder JL (2018). Diagnosis and treatment of urinary tract infections across age groups. Am J Obstet Gynecol.

[21] Zegers-Hochschild F, Adamson GD, Dyer S, Racowsky C (2017). The international glossary on infertility and fertility care, 2017. Fertil Steril.

[22] Dyer S, Chambers GM, de Mouson J (2016). International Committee for Monitoring Assisted Reproductive Technologies world report: assisted reproductive technology 2008, 2009 and 2010. Hum Reprod.

[23] Berkovitz A, Biron-Shental T, Pasternak Y (2017). Predictors of twin pregnancy after ovarian stimulation and intrauterine insemination in women with unexplained infertility. Hum Fertil (Camb).

[24] Calhaz-Jorge C, De Geyter C, Kupka M, European IVF-monitoring Consortium (EIM); European Society of Human Reproduction and Embryology (ESHRE) (2017). Assisted reproductive technology in Europe, 2013: results generated from European registers by ESHRE. Hum Reprod.

[25] Loftin RW, Habli M, Snyder C (2010). Late preterm birth. Rev Obstet Gynecol.

[26] Jacob J, Lehne M, Mischker A (2017). Cost effects of preterm birth: a comparison of health care costs associated with early preterm, late preterm, full-term birth in the first 3 years after birth. Eur J Health Econ.

[27] Patel RR, Steer P, Doyle P, Little MP, Elliott P (2004). Does gestation vary by ethnic group? A London-based study of over 122,000 pregnancies with spontaneous onset of labor. Int J Epidemiol.

[28] Steer P (2005). The epidemiology of preterm labor. BJOG.

[29] Saccone G, Zullo F, Roman A (2019). Risk of spontaneous preterm birth in IVF-conceived twin pregnancies. J Matern Fetal Neonatal Med.

[30] Khrouf M, Slimani S, Khrouf MR (2017). Progesterone for luteal phase support in in vitro fertilization: comparison of vaginal and rectal pessaries to vaginal capsules: a randomized controlled study. Clin Med Insights Womens Health.

[31] Van der Linden M, Buckingham K, Farquhar C, Kremer JA, Metwally M (2015). Luteal phase support for assisted reproduction cycles. Cochrane Database Syst Rev.

[32] Aghsa MM, Rahmanpour H, Bagheri M, Davari-Tanha F, Nasr R (2012). A randomized comparison of the efficacy, side effects and patient convenience between vaginal and rectal administration of Cyclogest (®) when used for luteal phase support in ICSI treatment. Arch Gynecol Obstet.

[33] Ioannidis G, Sacks G, Reddy N (2005). Day 14 maternal serum progesterone levels predict pregnancy outcome in IVF/ ICSI treatment cycles: a prospective study. Hum Reprod.

[34] Chakmakjian ZH, Zachariah NY (1987). Bioavailability of progesterone with different modes of administration. J Reprod Med.

[35] Henningsen AK, Pinborg A (2014). Birth and perinatal outcome and complications for babies conceived following ART. Semin Fetal Neonatal Med.

[36] Simoes T, Queros A, Marujo AT, Valdoleiros S, Silva P, Blickstein I (2015). Outcome of monochorionic twins conceived by assisted reproduction. Fertil Steril.

[37] Cetingoz E, Cam C, Sakalli M, Karateke A, Celik C, Sancak A (2011). Progesterone effects on preterm birth in high-risk pregnancies: a randomized placebo-controlled trial. Arch Gynecol Obstet.

[38] Serra V, Perales A, Meseguer J (2013). Increased doses of vaginal progesterone for the prevention of preterm birth in twin pregnancies: a randomized controlled double-blind multicenter trial. BJOG.

[39] Brizot M, Hernandez W, Liao A (2015). Vaginal progesterone for the prevention of preterm birth in twin gestations: a randomized placebo-controlled double-blind study. Am J Obstet Gynecol.

[40] El-Refaie W, Abdelhafez MS, Badawy A (2016). Vaginal progesterone for prevention of preterm labor in asymptomatic twin pregnancies with sonographic short cervix: a randomized clinical trial of efficacy and safety. Arch Gynecol Obstet.

[41] Brubaker SG, Pessel C, Zork N, Gyamfi-Bannerman C, Ananth CV (2015). Vaginal progesterone in women with twin gestations complicated by short cervix: a retrospective cohort study. BJOG.

[42] Biggio JR (2015). Short cervix and twins: progesterone, yes or no?. BJOG.

[43] Schuit E, Stock S, Rode L (2015). Global obstetrics network (GONet) collaboration. Effectiveness of progestogens to improve perinatal outcome in twin pregnancies: an individual participant data meta-analysis. BJOG.

[44] Durnwald CP, Momirova V, Rouse DJ (2010). Eunice Kennedy Shriver National Institute of Child Health and Human Development maternal-fetal medicine units network. Second trimester cervical length and risk of preterm birth in women with twin gestations treated with 17-α hydroxyprogesterone caproate. J Matern Fetal Neonatal Med.

[45] Lim AC, Schuit E, Papatsonis D (2012). Effect of 17-α hydroxyprogesterone caproate on cervical length in twin pregnancies. Ultrasound Obstet Gynecol.

[46] Senat MV, Porcher R, Winer N (2013). Groupe de Recherche en Obstétrique et Gynécologie. Prevention of preterm delivery by 17 alph0hydroxyprogesterone caproate in asymptomatic twin pregnancies with a short cervix: a randomized controlled trial. Am J Obstet Gynecol.

[47] Agra IKR, Carvalho MHB, Hernandez WR, Francisco RPV, Zugaib M, Brizot ML (2017). The effect of prenatal vaginal progesterone on cervical length in on selected twin pregnancies. J Matern Fetal Neonatal Med.

[48] Kushnir VA, Barad DH, Albertini DF, Darmon SK, Gleicher N (2017). Systematic review of worldwide trends in assisted reproductive technology 2004-2013. Reprod Biol Endocrinol.

